# Plant-Based Proteins, Peptides and Amino Acids in Food Products Dedicated for Sportspeople—A Narrative Review of the Literature

**DOI:** 10.3390/nu16111706

**Published:** 2024-05-30

**Authors:** Kinga Kostrakiewicz-Gierałt

**Affiliations:** Department of Tourism Geography and Ecology, Institute of Tourism, Faculty of Tourism and Recreation, University of Physical Education in Kraków, Jana Pawła II 78, 31-571 Kraków, Poland; kinga.kostrakiewicz@awf.krakow.pl

**Keywords:** activity, invention, nutrition, patent, plant, sport, survey

## Abstract

Plant proteins are increasingly seen as critical nutrient sources for both amateur and professional athletes. The aim of the presented study was to review the inventions and experimental articles referring to the application of plant-based proteins, peptides and amino acids in food products dedicated to sportspeople and published in the period 2014–2023. The literature search was conducted according to PRISMA statementsacross several key databases, including Scopus and ISI Web of Science. Altogether, 106 patents and 35 original articles were found. The survey of patents and inventions described in the articles showed the use of 52 taxa (mainly annual herbaceous plants), creating edible seeds and representing mainly the families *Fabaceae* and *Poaceae*. The majority of inventions were developed by research teams numbering from two to five scientists, affiliated in China, The United States of America and Japan. The greatest number of inventions applied plant-based proteins (especially protein isolates), declared the nutritional activity and were prepared in liquid or solid consistency. According to the reviewed studies, the intake of soybean and potato proteins might provide better results than animal-based protein (excluding resistance training), whereas the consumption of pea and rice protein does not possess any unique anabolic properties over whey protein. The analysis of other investigations demonstrated the varied acceptability and consumption of food products, while the high rating of the tested food products presented in four articles seems to be an effect of their sensual values, as well as other elements, such as production method, health benefits and cost-effectiveness. Considering the great potential of useful plant species, it might be concluded that future investigations focusing on searching for novel plant protein sources, suitable for the preparation of food products dedicated to amateur and professional sportspeople, remain of interest.

## 1. Introduction

Proteins are composed of naturally occurring 20 types of amino acids and are an essential component of human nutrition. Nevertheless, animal species (including humans) can produce solely 11 non-essential amino acids (NEAAs) represented by alanine, arginine, asparagine, aspartic acid, cysteine, glutamic acid, glutamine, glycine, proline, serine and tyrosine. The remaining essential amino acids (EAAs), represented by histidine, isoleucine, leucine, lysine, methionine, phenylalanine, threonine, tryptophan, and valine, must be obtained in the diet. At the same time, it should be pointed out that three branched-chain amino acids (BCAAs)-leucine, isoleucine, and valine—are unique among the EAAs due to their roles in neural function, blood glucose and insulin regulation, as well as protein metabolism. Plotkin et al. [1] argued that leucine, valine and isoleucine boost muscle growth and enhance exercise performance.

At the same time, Kreider and Bill [2] pointed out that there is a significant body of evidence to indicate that individuals who are engaged in intense training require more dietary protein than their sedentary counterparts. For most individuals, this level of protein intake can be obtained from a diet or dietary supplements. The International Society of Sports Nutrition (ISSN) confirmed that dietary supplements containing essential amino acids [3] and proteins [4] have beneficial effects on skeletal muscle maintenance and performance and should be used by athletes to achieve the exercise and training daily demands. To date, a substantial number of the narrative and systematic reviews of the literature have evidenced the important role of such supplementation in the diet of professional and recreative sportspeople. Some authors documented the favourable effects of proteins consumed as dairy products or as supplements in isolated or concentrated form on athlete performance [5,6]. The investigations of other researchers concentrated on the effects of protein supplementation on muscle mass and strength [7,8]. In turn, König et al. [9] pointed out that bioactive peptides could have a positive impact on changes in body composition and muscular performance, reduction of muscle damage following exercise, and inducing beneficial adaptions within the connective tissue. Similarly, other authors [10,11] discussed the role of protein hydrolysates providing mainly di- and tripeptides in muscle protein anabolism, exercise performance and muscle glycogen resynthesis. Furthermore, numerous researchers outlined the influence of the amino acids both: essential, e.g., [5,12,13] and non-essential, e.g., [5,14,15] on immune function, physical performance and mental health of sportspeople.

At the same time, it should be mentioned that the metabolic response to protein, peptide and amino acid administration has a high degree of interindividual variability and may remarkably depend, among others, on gender [16,17], sports discipline [16,18,19], diet type [20], as well as time of intake [21,22,23].

Over the last decades, the main sources of proteins in athlete’s diets were conventional products such as eggs, milk, whey, cheese and meat derived from terrestrial animals [24]. The whey protein obtained as a by-product in the production of cheese and butter is very frequently consumed as a supplement in sports nutrition due to a notable content of essential amino acids and BCAAs. In addition, whey proteins have shown several biological properties, among others antioxidant, anticancer, antidiabetic, anti-obesity or cardioprotective. The next widely used protein is ovalbumin showing a complete amino acid profile and excellent digestibility. Furthermore, a high-quality protein source providing all of the essential amino acids in adequate amounts and ratios approximately to human metabolic needs is considered to be the meat of livestock and poultry. At the same time, Penggalih et al. [25] pointed out that marine-derived proteins and peptides, due to their unique amino acid composition, bioavailability and bioactive properties, are a promising source of nutraceuticals and functional food ingredients. Bioactive peptides especially rich in branched-chain amino acids (BCAAs) have a positive effect on body composition, increase lean body mass and muscle strength, enhance glucose intake into muscles, as well as help to heal muscle soreness and recovery from heavy exercise. It is worth mentioning that among the most promising marine protein sources are listed, among others, tunicate, bivalve and fish. Nevertheless, recently, other promising alternative protein sources possible to use in modern sports nutrition such as insects [26,27,28,29], fungi [28,29,30] or algae [25,27,28,29] have emerged, and the published scientific data on their nutritional value is still on the rise. At the same time, it should be stated that proteins widely applicable in the diet of athletes might also derive from plants, considered as a sustainable source of bioactive peptides [31]. Nevertheless, as stated by Sá et al. [32], depending on the source, plant peptides may be deficient in some essential amino acids. Cereals contrary to pseudocereals contain low levels of lysine, while legumes have a deficiency in sulphur amino acids such as methionine and cysteine. To date, the use of proteins, peptides and amino acids was confirmed above all in the case of: *Avena sativa* L. [33], *Cicer arietinum* L. [34], *Glycine max* (L.) Merr. [35,36,37,38,39], *Ipomoea batatas* L. Lamarck [40], *Lens culinaris* Medik. [34], *Oryza sativa* L. [30,38], *Phaseolus lunatus* L. [34], *Phaseolus vulgaris* L. [34,41], *Pisum sativum* L. [30,34], *Triticum* L. [33,38], *Vigna radiate* [34] and *Zea mays* L. [42]. 

However, despite growing scientific interest in the application of plant-derived proteins, peptides and amino acids in sports nutrition, the current state of knowledge remains insufficient. Among others, there is a lack of comparative research presenting the use of particular plant taxa in food products dedicated to sportspeople, the activity of proteins deriving from different plant species, and the attitude of athletes towards food products containing protein of different plant species origin. Taking into account the insufficient state of knowledge, the presented study was undertaken. The specific aims were the following:

1. To prepare a survey of plant taxa applied in food products dedicated to sportspeople;
2. To make an overview of food products containing plant-based proteins, peptides and amino acids dedicated to sportspeople;
3. To assess the influence of particular species-deriving proteins, peptides and aminoacids on the health, body composition and performance of sportspeople;
4. To evaluate the frequency of consumption and acceptance of products containing plant-based proteins, peptides and amino acids by sportspeople.

## 2. Materials and Methods

### 2.1. Literature Search

For this survey, a systematic approach for synthesising information through a dedicated stepwise process for selecting the available peer-reviewed literature sources was applied. 

The literature search was conducted across several key databases, including Scopus and ISI Web of Science, most widely used for bibliometric analyses [43], as well as Google Scholar engine. Patents were searched by browsing the Google Patents and Espacenet Patent Search engines, gathering the largest number of open access patents [44,45]. The survey of literature records published from 1 January 2014 up to 31 December 2023 was carried out according to PRISMA statements with a focus on factorial combinations of the following keywords in the searches: (“plant”) and (“amino acids” or “peptides” or “proteins”) and (“athlete” or “sport”) and (“food” or “nutrition” or “diet”).

The selection terms were examined from the title, abstract and keywords. The literature search was conducted from 5 January to 20 February 2024. The results included 1240 hits from the ISI Web of Science, 443 hits from Scopus,1687 from Google Scholar, 1601 from Google Patents, and 4324 from Espacenet. After the manual removal of grey literature (posts in blogs, letters, manuals, guides, bulletins, newsletters, editorials, commentaries, theses, dissertations, reports, conference proceedings and meeting notes) from the lists of searches, the patents and peer-reviewed articles were selected. Following the removal of duplicates (publications indexed in more than one database), the abstracts of patents and articles were screened for relevance and eligibility. 

### 2.2. Study Eligibility and Selection

During abstract and full-text screening, the inclusion criteria for patents were: (i) the use of plant-based proteins, peptides and amino acids in invented food products suitable for sports practitioners, and (ii) abstract and full-text were written in English. 

**Figure 1:**
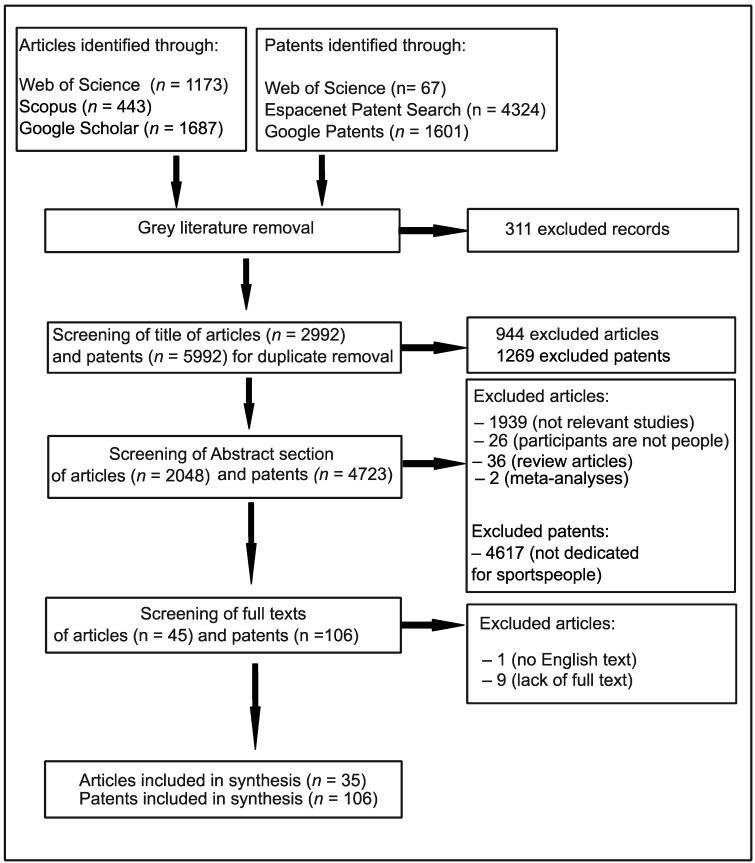
The procedure for the literature search.

The inclusion criteria for articles were as follows: (i) the investigations were relevant to application of plant-based proteins, peptides and amino acids in sport nutrition, (ii) the investigations included humans as participants, (iii) the investigations were observational, descriptive studies (case report/case series), observational, analytical studies (case–control studies, cross-sectional studies, cohort studies) or experimental studies (randomised controlled trials), (iv) there were no limits regarding the age, weight, sex, nationality or number of participants, (v) there were no limits in geographical location or time period of the investigations, and (vi) the abstract and full text were written in English. The exclusion criteria for articles were as follows: (i) lack of available full-text version, (ii) the investigations were meta-analyses, or (iii) systematic reviews. A chart detailing the search results is presented in Figure 1.

To assess the quality of the included studies and reduce the potential for misclassification, the abstracts and then full texts of patents and articles were subjected to critical double screening. From eligible patents and articles the following data were extracted: author names, author number, affiliation of first author, year of publication, title and characteristic of invention (consistency, form, plant species used as source of proteins), as well as additionally the age, gender, nationality of participants and outcomes of study in the case of articles. The aforementioned data were extracted using the form created in Microsoft Excel. 

The statistical significance of differences in the number of inventions: (i) developed by different number of authors, (ii) applying various constituents (amino acids, peptides, proteins), (iii) showing diverse activity (nutrition, increase in muscle mass and strength etc.), and (iv) presenting varied consistency (liquid, solid, etc.), was checked using non-parametric Kruskal–Wallis H test. 

## 3. Results

### 3.1. An Overview of Taxa Recorded in the Inventions

In total, 52 taxa including 45 species representing 20 botanical families were mentioned in the descriptions of presented inventions (Table 1). The majority of taxa represented the families *Fabaceae* (pulses) and *Poaceae* (cereals). Soybean *Glycine max* L. Merr., pea *Lathyrus oleraceus* Lam., rice *Oryza sativa* L., maize *Zea mays* L., as well as common wheat *Triticum aestivum* L., belong to the most frequently applied species. Also, the family *Amaranthaceae* (pseudocereals) was abundantly represented. In total, the species pool mentioned in the descriptions of surveyed products dedicated to sportspeople consisted of 24 (53.3%) annuals, 17 (37.8%) perennials, 1 (2.2%) species with lifespan ranges from one to two years, and 3 (6.7%) species showing annual or perennial lifespan. The number of herbaceous plants reached 35 (77.8%), the number of shrubs amounted to 3 (6.7%), and the number of trees was 4 (8.9%). Moreover, 3 (6.7%) species might be classified as shrubs or trees (depending on the height of the individual species). The species applied in products for sportspeople produce at least one edible organ such as seeds (38 species), fruits(23 species), leaves (23 species), shoots (7 species), roots and tubers (5 species), as well as inflorescences, apical buds and leaf petioles (1 species). 

### 3.2. An Overview of the Inventions

**Figure 2:**
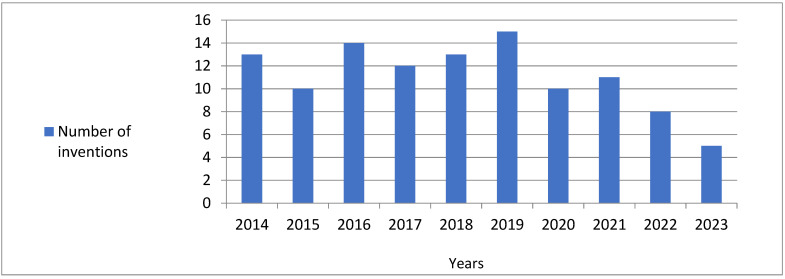
The number of inventions referring to products suitable for sports nutrition containing plant-based amino acids, peptides and/or proteins developed in the period 2014–2023.

**Figure 3:**
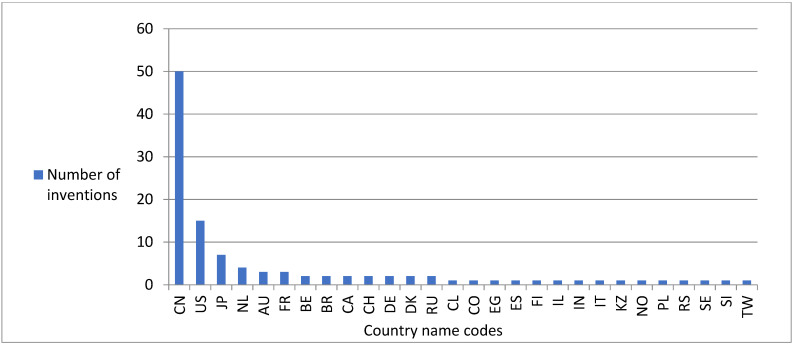
The total number of inventions referring to products suitable to sports nutrition containing plant-based amino acids, peptides and/or proteins developed in the period 2014–2023 by authors affiliated with Australia (AU), Belgium (BE), Brazil (BR), Canada (CA), China (CN), Colombia (CO), Denmark (DK), Egypt (EG), Finland (FI), France (FR), Germany (DE), India (IN), Israel (IL), Italy (IT), Japan (JP), Kazakhstan (KZ), The Netherlands (NL), Norway (NO), Poland (PL), The Russian Federation (RU), Serbia (RS), Slovenia (SI), Spain (ES), Sweden (SE), Switzerland (CH), Taiwan (TW), and The United States (US).

Altogether, 106 patents and 5 original articles issued in the years 2014–2023 were noted. The number of inventions developed in particular years ranged from 5 to 15, and achieved the greatest value in the year 2019, while in the subsequent years, it decreased (Figure 2). Altogether, the authors were affiliated with 28 countries. The greatest number of inventions was developed by researchers from China, the United States of America and Japan; a lower number of inventions was obtained by scientists represented from other countries such as The Netherlands, Australia and France; while the lowest number of inventions was presented by authors from Belgium, Brazil, Canada, Switzerland, Germany, Denmark, The Russian Federation, Colombia, Egypt, Spain, Finland, Israel, India, Italy, Kazakhstan, Norway, Poland, Serbia, Sweden, Slovenia and Taiwan (Figure 3). Altogether, 18 inventions were developed by one author, while 93 invention investigations were developed in research teams numbering from two to ten scientists. The value of the Kruskal–Wallis H test evidenced that the number of inventions created by research teams consisting of more than six authors was remarkably lower than those created by one author or developed in teams numbering from two to five scientists (Table 2). 

The value of the H Kruskal–Wallis test showed that plant-based amino acids and peptides were applied in a lower number of inventions as compared to proteins. At the same time, it should be mentioned, that the application of plant-based protein isolate was declared in 36 inventions, the use of protein concentrate was found in 10 inventions, whereas the application of protein hydrolysate was only noted in 3 inventions (see Table A1, Table A2, Table A3, Table A4 and Table A5). Regarding the activity of inventions, the value of the Kruskal–WallisHtest indicated that the majority of them was devoted to nutrition, an appreciable number of them were aimed at the increase in muscle mass and strength, while a significantly lower number of products provided: (i) the improvement mental and physical health, (ii) the relieving of fatigue and muscle recovery, (iii) the increase in performance and/or endurance, and (iv) the control of body weight. The value of the Kruskal–Wallis H test confirmed that the majority of inventions were prepared in liquid consistencies(e.g., beverages, drinks, emulsions) or solid consistencies (e.g., bars, snacks, cakes). The lower number of inventions were prepared in bulk solid consistency (e.g., powders, granules), whilst the lowest number of the inventions were prepared in semi-liquid-consistency (e.g., porridge, slurry, pulp) or semi-solid-consistency (e.g., cream, gel, jam, yoghurt).

### 3.3. An Overview of Original Scientific Articles

In total, 18 publications were devoted to investigations of the effects of plant-based peptide and protein supplementation on sportspeople’s health, performance and body composition (Table 3). The majority of authors investigated the effects of peptides and proteins derived from soybean, whereas the number of investigations focused on the effects of pea, rice, maize, amaranth, potato and wheat was lower. 

Several studies indicate that soy protein boosts muscle mass, strength and recovery. For instance, Jiang [158], Shenoy et al. [159], Amare and Chekol [160], Lynch et al. [161], Kritikos et al. [162] and Jin et al. [163] observed improvements in muscle recovery and strength, while Röhling et al. [164] noticed an increase in lean mass weight, suggesting a beneficial role for soy peptides in sports nutrition. On the other hand, Reidy et al. [165] and Hevia-Larraín et al. [166] noted minimal effects on muscle adaptations especially to resistance training, which highlights the variability in the responses. 

Other researchers [167,168,169,170] demonstrated that ingestion of whey and pea protein produces similar outcomes in measurements of body composition, muscle thickness, force production and strength. Moon et al. [171] stated that intake of rice or whey protein in combination with an eight-week resistance training programme led to similar changes in body composition and performance outcomes. Saracino et al. [172] observed that pre-sleep ingestion of rice and pea proteins as well as whey isolate, or hydrolysate did not aid in muscle recovery when damaging eccentric exercise was performed in the morning. However, it is worth mentioning that Pinckaers et al. [173] documented that the ingestion of potato protein concentrates in comparison to the intake of milk protein increases muscle protein synthesis rates, both at rest and during recovery from exercise.

The other authors compared commercially available drinks with maize juice [174] and amaranth-based beverages [175] and stated that plant drinks are very effective in supporting optimal performance and hydration.

In total, 12 articles referred to the popularity of products containing plant-based proteins in sportspeople (Table 4). The high acceptability and substantial consumption of plant-based protein products by athletes was noticed by Shaw et al. [176], Śliż et al. [177], as well as Jakše and Jakše [178]. In turn, investigations concentrated solely on female athletes showed a positive relation between considerable consumption of plant-based proteins and lack of PMS-related athletic impairment [179], as well as among substantial dietary intake of vegetable protein and menstrual dysfunction [180]. Other investigations showed low interest of sportspeople in the consumption of plant-based proteins. Gillen et al. [181] stated that the plant-based protein sources represented by bread, cereals and grains, vegetables and fruits, as well as cakes and biscuits are consumed less frequently than animal-based protein. McDaid et al. [182] documented the intake of plant proteins solely by amateur athletes. Franca et al. [183] noted that adolescent athletes ingest mainly animal-derived protein, particularly meat and eggs, while plant-based protein is rather rarely eaten.

In addition, four publications referred to testing and evaluation of the activity of particular products dedicated to sportspeople (Table 5). The high rating received for Soy Protein Isolate Enriched Pregame Chocolate Bar [184], millet bar [185], maize recovery beverage [186] and high protein pretzels containing soy and wheat ingredients [187]. The nutrient content and quality of all the presented products were appreciated by consumers. Moreover, in the case of selected products, other benefits such as the possibility of consumption by lactose intolerant active people or low cost of preparation were pointed out.

## 4. Discussion

### 4.1. An Overview of Taxa Recorded in the Inventions

According to the conventional point of view, animals compared with plant-food proteins are considered the crucial source of a variety of bioactive peptides influencing many physiological responses and the maintenance of human good health conditions [188]. Nevertheless, the performed investigations presenting the use of 52 plant taxa in alimentary products dedicated to sportspeople seem to not support these statements. The performed survey of inventions dedicated to sportspeople proved that the majority of species mentioned in the patent descriptions belong to annual herbaceous plants representing the families *Fabaceae* and *Poaceae* creating edible seeds showing substantial nutritional value and playing a considerable role in global food security [189,190,191,192,193]. At the same time, it is worth mentioning that this finding partly supports the study of Murowaniecki Otero [29], who pointed out that the main alternative protein sources among plants are represented by species from the *Fabaceae* and *Asteraceae* families. Moreover, it should be mentioned that due to the significant content of bioactive peptides and proteins seeds of chia [194], flax [194], pumpkin [194,195], watermelon [194,196] and amaranth [194], are also frequently used in food production, especially for sportspeople. At the same time, the reviews of the literature conducted by Arbach et al. [197] and Qamar et al. [198] evidenced the extensive application of seeds of numerous species as a source of proteins in plant-based beverages. The performed investigations showing that numerous plant species used in surveyed patents create edible fruits correspond with the findings proving that the seed pods of peas and beans [199], the berries of goji [200], as well as the drupes of date [201] are rich sources of protein using frequently as food ingredients. Moreover, the conducted investigations demonstrating that many species create edible leaves are consistent with the findings of Hadidi et al. [202], who reviewed the nutritional value of green leaf proteins, among others, in alfalfa, amaranth, moringa and spinach. Others worth mentioning as protein sources observed in the patent descriptions are underground organs, such as the tuberous roots of the sweet potato and the tubers of the Chinese yam and potato. Chandrasekara and Kumar [203] highlighted the immense potential of the aforementioned organs as functional foods and nutraceutical ingredients in disease risk reduction and wellness. 

### 4.2. An Overview of the Inventions

The greatest number of inventions published in the year 2019 and their decrease in the subsequent years corresponds with the tendency presented by Arbach et al. [197], who investigated the number of patents referring to plant-based beverages developed in the years 2015–2020. The aforementioned authors documented a similar number of patents developed in the years 2015–2018, then a substantial increase in the year 2019, and a considerable decrease in the year 2020. A similar tendency was found by Murowaniecki Otero et al. [29], who explored the annual evolution of scientific articles and patents developed in the years 1998–2021 and referred to alternative protein sources. Moreover, the low number of inventions observed in the presented investigations during the year 2023 compared to the previous years may be due to the fact of waiting for indexation in the databases. It is worth mentioning that in the database Espacenet, the publication of an invention appears18 months after the filing date [204]. A similar trend of the decline in the number of patents in the last years of the review period was already noticed in the case of the review of nutritional products for sportspeople based on the kidney bean [41] and sweet potato [40]. On the other hand, the survey of the use of maize [42] and soybean [37] showed a gradual increase in the number of inventions in the consecutive study periods. 

The performed observations, showing that the majority of inventions containing plant proteins were developed by researchers affiliated in China, The United States of America and Japan, correspond with other findings referring to the application of plant sources in food products dedicated to sportspeople. The greatest number of authors who developed inventions based on constituents deriving from soybeans were affiliated with The United States of America, China and Japan [37]. Moreover, researchers affiliated with The United States of America and China published the greatest number of patents containing constituents derived from kidney beans [42]. The substantial interest in the development of inventions containing plant proteins by scientists affiliated with China and The United States of America is not surprising considering that the aforementioned countries have for many years belonged to the world’s leading crop producers [205]. 

The performed investigations showing that the majority of inventions were developed by researcher teams numbering from 2 to 10 authors confirm the global trend of the transformation of scientific research in numerous disciplines of natural and social sciences from individual research to teamwork [206]. At the same time, the obtained results evidenced that the number of inventions created by research teams consisting of more than six cooperating authors was remarkably lower than those created by a minor number of scientists. 

The performed investigations showed that plant-based amino acids and peptides were applied in a lower number of inventions as compared to proteins. At the same time, it should be mentioned that the application of plant-based protein isolate was declared in a majority of inventions. Such findings correspond with the studies of Sharif et al. [207], who pointed out that legumes, cereals and oilseeds are ideal sources of protein for extraction concentrates and isolates, showing different functional attributes depending on the raw materials and extraction techniques. They added that the most widely used species include, among others, soybean, pea, lupin, chickpea, wheat, rice, maize, barley, sorghum, canola and sunflower. In addition, the performed study shows that the majority of inventions present nutritional activity corresponding with surveys of food products dedicated to sportspeople and based on constituents derived from kidney beans [41] and maize [42]. Simultaneously, the aforementioned findings appear to be concordant with the survey of the literature conducted by Munialo [208], evidencing the considerable nutritional value of plant proteins used in the food industry. The appreciable number of patents aiming to increase muscle mass and strength, as noted in the presented studies, might be linked with the augmenting demand for such products by amateur and professional athletes. The close relationship between muscle mass and strength and overall athlete performance was evidenced, among others, by Suchomel et al. [209]. The lower number of inventions devoted to the improvement of mental and physical health, the relieving of fatigue and muscle recovery, the increase in performance and/or endurance, and the control of body weight corresponds with other studies [37,40,41,42]. 

Considering the consistency of the inventions, the obtained results show that their number decreases significantly from liquid or solid consistency via bulk solid, to semi-liquid or semi-solid. These findings partly support the research of Cui et al. [210], who argued that the sports food market contains sports drinks and solid and semi-solid sports food. The significant number of products with a solid consistency might be linked to advantages such as quick satisfaction of appetite after intake and perception of satiety. It is worth mentioning, that the wide application of plant-derived protein in many products presenting solid consistency, such as cakes, cookies, muffins, bread, pasta, snacks, bars and noodles, was documented regarding pulses [34,211,212], cereals [213] and pseudocereals [212,213].The substantial number of inventions in liquid consistency might be connected with the fact that this is the most attractive and desirable due to their convenience, and the possibility to meet consumer demands for, among others, contents and size, as well as for easy distribution and storage [214]. Moreover, the considerable popularity of liquid consistency might be linked to the substantial number of patents with bulk solid consistency, given that numerous beverages are made in the form of a powder, as stated by Arbach et al. [197]. 

### 4.3. An Overview of the Original Scientific Articles

According to many authors, e.g., [215,216,217] most plant-based proteins present lower anabolic properties than animal-based proteins. This phenomenon is connected with excess content of essential amino acids and often deficiency in one or more specific amino acids (such as lysine, methionine and tryptophan), as well as lower digestibility, compared to animal-based sources. Therefore, the aforementioned authors stated that the ingestion of plant-derived proteins results in a lower muscle protein synthesis in comparison to the ingestion of an equivalent amount of animal-derived protein. Nevertheless, the reviewed clinical trials evidenced the favourable effects of potato and soybean proteins and peptides compared to whey and milk proteins on the increase in strength of muscles, delay of onset of muscle soreness, mitigation of exercise-induced muscle stress reactions, as well as muscle recovery. At the same time, the performed investigations support the findings of Messina et al. [218], who evidenced the lack of difference between the effects of soy protein versus animal protein on gains in muscle mass and strength in response to resistance exercise. Moreover, the presented review of the literature sources proved that the consumption of pea and rice protein does not possess any unique anabolic properties over whey protein.

The varied acceptability and intake of food products containing plant proteins by sportspeople might be connected with food habits, as well as traditions in national cuisines. The low intake of plant proteins by sportspeople from Ireland and The Netherlands does not surprise and reflects Western dietary habits, including the high intake of animal-based proteins such as red meat Hone et al. [219] documented that meat and dairy had the largest percentage contribution to total protein intake across both sexes and all age groups of Irish adults. Similarly, the low consumption of plant-based protein by Brazilian sportspeople corresponds with the significant tendency to a gradual increase in animal protein consumption in the period 1961–2011 [220], as well as the strong progressive reduction of bean consumption in Brazil documented by Granado et al. [221]. The aforementioned authors projected that by 2025 regular consumption of beans will stop being the predominant habit in the country. On the other hand, the substantial acceptance of plant-based protein sources by Polish athletes is not consistent with a general preference for meat products presented in the survey conducted in households [222]. The substantial consumption of plant-based proteins declared by sportspeople from Canada and The United States of America does not support the findings of Auclair and Burgos [223], as well as Hoy et al. [224], who documented the low consumption of plant protein in adult populations. At the same time, the increasing intake of plant protein, especially cereals by Canadians as recorded by Marinangeli et al. [225] should be mentioned.

The performed survey of the literature evidenced the high rating of tested food products by sportspeople. The high rating of maize beverage noted in the performed investigations is consistent with the observations of Ortiz-Solà et al. [226], who evidenced the acceptability of drinks containing hazelnuts, coconut, grains and legumes and their use as a replacement for animal-based beverages and covering nutritional demand. The high rating of food products containing soy proteins, such as pretzels and bars, observed in the performed literature review might suggest that the volatile and non-volatile bean off-flavours [227] were mitigated or removed during the processing process. According to Saint-Eve et al. [228] the unpleasant flavour and displeasing mouthfeel perception of flour from legumes in food products may be a substantial barrier to their acceptance. The high rating of bars containing millet flour seems to correspond with the findings of other authors evidencing the high popularity of cereal bars containing millet flour among consumers, e.g., [229,230]. As the performed survey shows, apart from the high sensory value of the tested products, the other properties such as preparation method, effects and cost-effectiveness were appreciated by athletes. Such findings seem to correspond with the investigations of Jeong and Lee [231], showing that the acceptability of foods and drinks is affected by numerous elements such as information on the health benefits of the product or ingredients and the content of food labels. 

According to Gregory et al. [232], due to its non-systematic nature, there are no formally established guidelines for conducting narrative reviews, which might result in potential biases in selection and often in qualitative syntheses of results. Considering this, it should be stated that although the review emphasises promising aspects of plant proteins, it is crucial to also consider the limitations observed in the studies, which include small sample sizes, short durations of dietary interventions, and limited diversity in study populations. Addressing these issues in future research will be vital to corroborate findings and generalise them to broader populations.

## 5. Conclusions

The performed investigations evidenced the use of 52 taxa representing mainly annual herbaceous plants creating edible seeds, especially from the families *Fabaceae* and *Poaceae*. The greatest number of inventions published in 2019 and its decrease in the subsequent years (especially in the last year of the study period), might be a consequence of the long time period needed for indexation in the databases. The substantial interest in the development of inventions by scientists affiliated with China and The United States of America might be connected to the fact that the aforementioned countries belong to the leading crop producers. The majority of investigations undertaken by the research teams confirm the global trend of the transformation of scientific research in numerous disciplines of sciences, from individual research to teamwork. The substantial number of inventions using plant proteins (compared to peptides and amino acids) correspond with the worldwide tendency of the numerous crops used in the production of protein ingredients in the form of defatted flour, concentrates and isolates.

The nutritional activity presented by almost all the inventions is consistent with the considerable health value of plant proteins used in the food industry, as evidenced in the literature. In turn, the appreciable number of patents aiming to increase muscle mass and strength might be linked with the augmenting demand for such products by amateur and professional athletes due to the evidenced close relationship between muscle mass and strength and overall athlete performance. The significant number of products with solid consistency might be linked with the quick satisfaction of appetite after intake, whereas the considerable number of inventions with liquid consistency might be connected with meeting consumer demands for contents, size, and the possibility of easy distribution and storage. The appreciable number of patents with bulk solid consistency might be linked to the popularity of liquid consistency and the preparation of beverages in the form of soluble powders. According to the reviewed investigations, the intake of soybean and potato proteins might provide better results than animal-based protein (excluding resistance training), whereas the consumption of pea and rice protein does not possess any unique anabolic properties over whey protein. The varied acceptability and consumption of food products containing plant proteins by sportspeople might be connected with national food habits or remain in opposition to traditional national cuisine. The high rating of tested food products by sportspeople is based on sensual values, as well as other elements such as production method, health benefits and cost-effectiveness. 

Considering the great potential of useful plant species shown in the presented review, it might be stated that further investigations seem to be strongly desirable. Their main direction should be focused on searching for novel, promising plant protein sources suitable for the preparation of food products dedicated to amateur and professional sportspeople.

## Figures and Tables

**Table 1:** The survey of taxa containing amino acids, peptides and/or proteins applied in inventions suitable to sports nutrition developed in the years 2014–2023. Abbreviations: Lifespan: A—annual, P—perennial, Life form: H—Herbaceous plant, S—shrub, T—tree. The dots mean lack of data.

Family	Taxon		Number of Inventions	References	Species Characteristics According to Fern [46]		
	Latin Name	Common Name					
					Lifespan	Life Form	Edible Uses
*Amaranthaceae*	*Amaranthus* L.	Amaranth	2	[47,48]	.	.	.
	*Chenopodium quinoa* Willd.	Quinoa	10	[48,49,50,51,52,53,54,55,56,57]	A	H	Seeds
	*Spinacia oleracea* L.	Spinach	2	[58,59]	A	H	Leaves, seeds
*Anacardiaceae*	*Pistacia vera* L.	Pistachio	1	[60]	P	T	Seeds
*Asteraceae*	*Helianthus annuus* L.	Sunflower	4	[61,62,63,64]	A	H	Leaf petioles, seeds, flower buds,
*Arecaceae*	*Cocos nucifera* L.	Coconut	2	[62,63]	P	T	Apical bud, inflorescence, fruit with seed, roots, seed
*Brassicaceae*	*Brassica napus* L.	Canola, rapeseed, colza	7	[54,64,65,66,67,68,69]	A or P	H	Leaves, immature flowering stems, seeds
	*Crambe* L.	Crambe	1	[63]	.	.	.
	*Moringa oleifera* Lam.	Horseradish Tree	2	[64,70]	P	S or T	Flowers, seed pods, seeds, seedlings, leaves, shoots
*Cannabaceae*	*Cannabis sativa* L.	Hemp	7	[50,60,63,66,71,72,73]	A	H	Leaves, seeds
*Convolvulaceae*	*Ipomoea batatas* (L.) Lam.	Sweet potato	2	[50,74]	P	H	Tuberous roots
*Cucurbitaceae*	*Citrullus lanatus* (Thunb.) Matsum. and Nakai	Watermelon	1	[60]	A	H	Fruits, leaves, seeds
	*Cucurbita pepo* L.	Pumpkin	2	[60,64]	A	H	Flowers, flower buds, fruits, leaves, seeds
*Dioscoreaceae*	*Dioscorea polystachya* Turcz.	Chinese yam	1	[75]	P	H	Fruits, tubers
*Euphorbiaceae*	*Plukenetia volubilis* L.	Sacha ichni	1	[76]	P	S	Leaves, seeds
*Fabaceae*	*Arachis hypogaea* L.	Peanut	8	[59,60,63,65,67,77,78,79]	A	H	Seeds, leaves, fruits
	*Cajanus cajan* (L.) *Millsp*	Pigeon pea	2	[80,81]	P	S	Seeds, fruits, leaves, shoots
	*Canavalia gladiata (Jacq.) DC.*	Sword bean	2	[80,81]	A or P	H	Seeds, fruits
	*Ceratonia siliqua* L.	Carob	1	[63]	P	S or T	Seeds, fruits
	*Cicer arietinum* L.	Chickpea	9	[55,63,64,69,76,80,81,82,83]	A	H	Seeds, fruits, shoots
	*Glycine max* L. Merr.	Soybean	70	[48,50,53,54,55,60,61,63,65,66,67,69,71,73,74,78,79,80,81,84,85,86,87,88,89,90,91,92,93,94,95,96,97,98,99,100,101,102,103,104,105,106,107,108,109,110,111,112,113,114,115,116,117,118,119,120,121,122,123,124,125,126,127,128,129,130,131,132,133,134,135]	A	H	Seeds, fruits, leaves
	*Lablab purpureus* (L.) Sweet	Hyacinth bean	2	[80,81]	P	H	Seeds, fruits, leaves, flowers, root
	*Lathyrus oleraceus* Lam.	Pea	35	[48,54,56,58,59,61,63,64,65,66,67,68,70,72,73,74,76,80,81,84,86,87,108,110,119,130,132,136,137,138,139,140,141,142]	A	H	Seeds, fruits, shoots, leaves
	*Lupinus* L.	Lupine	5	[62,63,64,76,137]	.	.	.
	*Medicago sativa* L.	Alfalfa	3	[63,64,137]	P	H	Seeds, shoots, leaves
	*Phaseolus vulgaris* L.	Kidney bean	13	[48,63,67,75,80,81,104,136,137,138,143,144]	A or P	H	Seeds, fruits, leaves
	*Prosopis* L.	Mesquite	1	[63]	.	.	.
	*Psophocarpus tetragonolobus* (L.) *DC.*	Goa bean	1	[60]	P	H	Seeds, fruits, leaves, shoots, roots
	*Tamarindus indica* L.	Tamarind	1	[63]	P	T	Seeds, fruits, leaves, flowers
	*Trifolium* L.	Clover	2	[63,137]	.	.	.
	*Vicia faba* L.	Fava bean	6	[64,80,81,83,108,136]	A	H	Seeds, fruits, leaves
	*Vicia lens* (L.) Coss. and Germ.	Lentil	7	[55,63,64,67,76,137,138]	A	H	Seeds, fruits
	*Vigna angularis* (Willd.) Ohwi and H. Ohashi	Red bean, Adzuki bean	2	[80,81]	A	H	Seeds, fruits
	*Vigna radiata* (L.) *R. Wilczek*	Mung bean	3	[64,80,81]	A	H	Seeds, fruits, leaves, shoots
	*Vigna unguiculata* (L.) Walp.	Cowpea	2	[80,81]	P	H	Fruits
*Juglandaceae*	*Juglans regia* L.	Walnut	4	[75,79,108,134]	P	T	Seeds, leaves
*Malvaceae*	*Gossypium* L.	Cotton	1	[63]	.	.	.
*Lamiaceae*	*Salvia hispanica* L.	Mexican chia	2	[62,64]	A	H	Seeds
*Linaceae*	*Linum usitatissimum* L.	Flaxseed	5	[56,60,64,66,145]	A	H	Seeds
*Papaveraceae*	*Papaver* L.	Poppy	1	[60]	.	.	.
*Pedaliaceae*	*Sesamum indicum* L.	Sesame	3	[60,63,145]	A	H	Seeds, leaves
*Poaceae*	*Avena sativa* L.	Oat	5	[48,65,110,137,146]	A	H	Seeds
	*Hordeum vulgare* L.	Barley	3	[74,110,137]	A	H	Seeds
	*Oryza sativa* L.	Rice	24	[54,55,56,64,65,66,67,70,72,73,75,76,86,87,89,113,118,119,130,132,137,142,147,148]	A or P	H	Seeds
	*Secale cereale* L.	Rye	2	[48,137]	A	H	Seeds
	*Sorghum bicolor* L. Moench	Sorghum	1	[137]	A	H	Seeds
	*Triticum aestivum* L.	Common wheat, bread wheat	20	[48,54,65,74,76,78,86,87,89,96,101,113,116,118,119,137,149,150,151,152]	A	H	Seeds
	*Zea mays* L.	Maize, sweet corn	22	[54,63,67,74,75,78,87,89,91,101,105,113,116,121,126,128,132,137,148,153,154,155]	A	H	Seeds, unripe cobs
*Polygonaceae*	*Fagopyrum tataricum* (L.) Gaertn.	Tartarian buckwheat	1	[156]	A	H	Seeds, leaves
*Rhamnaceae*	*Ziziphus jujuba* Mill.	Red date, Chinese date, Chinese jujube, jujube	1	[134]	P	S or T	Fruits, leaves
*Solanaceae*	*Lycium barbarum* L.	Goji, matrimony vine	2	[70,73]	P	S	Fruits, leaves
	*Solanum tuberosum* L.	Potato	5	[61,64,74,94,157]	P	H	Tubers

**Table 2:** The total number of inventions referring to products suitable to sports nutrition developed in the period 2014–2023 by a varied number of authors, applying different constituents, showing diverse activity and presenting different consistency. The different letters in superscript mean statistically significant differences. Asterisks mean the statistical significance level *** *p* < 0.001.

		Years										Mean (±SD)	Kruskal–Wallis Htest
		2014	2015	2016	2017	2018	2019	2020	2021	2022	2023		
Number of authors	1	2	1	4	5	1	2	1	1	0	1	1.8 (±1.5) ^a^	H = 43.1 ***
	2	2	3	7	2	0	4	2	2	0	0	2.2 (±2.1) ^a^	
	3	3	3	4	1	5	2	5	2	4	0	2.9 (±1.7) ^a^	
	4	0	1	0	0	2	3	1	1	3	2	1.3 (±1.2) ^a^	
	5	4	1	0	3	3	2	0	2	1	1	1.7 (±1.3) ^a^	
	6	0	0	0	1	1	0	1	0	0	0	0.3 (±0.5) ^b^	
	7	2	0	1	0	0	1	0	0	0	1	0.5 (±0.7) ^b^	
	8	0	0	0	0	1	1	0	1	0	0	0.3 (±0.5) ^b^	
	9	0	1	0	0	0	0	0	1	0	0	0.2 (±0.4) ^b^	
	10	0	0	0	0	0	0	0	1	0	0	0.1 (±0.3) ^b^	
Constituents	Proteins	11	8	8	7	11	11	8	9	8	4	8.5 (±2.2) ^a^	H = 23.0 ***
	Peptides	2	3	6	6	3	4	4	2	0	2	3.2 (±1.9) ^b^	
	Amino acids	1	0	1	0	2	2	1	0	0	0	0.7 (±0.8) ^b^	
Activity	Nutrition	8	8	7	11	14	15	5	6	8	4	8.6 (±3.7) ^a^	H = 34.3 ***
	Increase in muscle mass and strength	7	2	5	1	3	4	0	3	1	1	2.7 (±2.2) ^ab^	
	Improvement in mental and physical health	2	2	2	4	2	2	3	3	0	1	2.1 (±1.1) ^b^	
	Relieving of fatigue and muscle recovery	2	2	2	4	1	3	2	2	1	1	2.0 (±0.9) ^b^	
	Increase in performance and/or endurance	1	3	1	0	0	2	2	2	0	1	1.2 (±1.0) ^b^	
	Control of body weight	0	0	0	1	3	0	0	1	0	0	0.5 (±1.0) ^b^	
Consistency	Liquid	5	3	10	9	7	8	6	4	4	2	5.8 (±2.7) ^a^	H = 26.1 ***
	Solid	7	5	6	2	6	9	9	7	3	4	5.8 (±2.3) ^a^	
	Bulk solid	6	3	8	4	3	2	3	3	2	0	3.4 (±2.2) ^ab^	
	Semi-solid	5	1	2	2	2	5	1	3	0	0	2.1 (±1.8) ^b^	
	Semi-liquid	1	0	1	0	4	0	1	1	1	0	0.9 (±1.2) ^b^	

**Table 3:** A review of original articles devoted to the effect of plant-based amino acids and proteins on sportspeople’s health, in chronological order. Abbreviations of country names: BR—Brazil, CN—China, DE—Germany, ET—Ethiopia, GR—Greece, IN—India, NL—The Netherlands, MX—Mexico, MY—Malaysia, PT—Portugal, US—the United States. The dot means lack of data.

References	Physical Activity	Country	Plant Species	Treatment	Results
Jiang [158]	Middle-distance runners	.	Soybean	Target: soybean peptides Control: glucose	Soybean peptide consumption: ↑ weight and lean body mass, serum testosterone ↓ serum creatine kinase (CK)
Shenoy et al. [159]	Boxing, cycling	IN	Soybean	Target: soybean protein Placebo: aspartame	Soybean protein consumption: ↑ muscle recovery, ↓ muscle damage, oxidative stress
Amare, Chekol [160]	Handball	ET	Soybean	Target: consumption of soybean protein (SP) vs. whey protein (WP) Control: mix of water, sugar and barley powder	Soybean protein consumption: ↑ maximal hand grip muscle strength, peak anaerobic power
Lynch et al. [161]	Recreational training	US	Soybean	Target: consumption of soybean protein isolate vs. whey protein isolate	Soybean protein consumption: ↑ total body mass, lean body mass, peak torque of leg extensors and flexors
Kritikos et al. [162]	Soccer	GR	Soybean	Target: soy protein vs. whey protein Placebo: maltodextrin	Soy consumption: mitigated ↓ speed running ↑ delayed onset of muscle soreness, creatine kinase, total antioxidant capacity, protein carbonyls ↓ glutathione ↑ recovery of protein carbonyls
Jin et al. [163]	Cycling	CN	Soybean Rice Wheat	Target: soybean, wheat and rice peptides Control: lack of peptides	Soybean, wheat and rice peptide consumption: ↑ total body triglyceride breakdown, non-esterified fatty acid uptake, fat oxidation
Röhling et al. [164]	Endurance athletics (marathon race)	DE	Soybean	Target: soy intake of a soybean protein-based supplement over a three-month period Control: lack of supplement	Soybean protein consumption: Mitigated ↑ of myoglobin, leukocytes, cortisol interleukins in blood
Reidy et al. [165]	Resistance exercise training	US	Soybean	Target: soybean-dairy protein blend vs. whey protein isolate Placebo: maltodextrin	Soybean protein consumption: ↑ lean body mass No differences among effects of consumption of plant- and animal- based proteins on leg muscle hypertrophy and vastus lateralis myofiber-type-specific cross-sectional area
Hevia-Larraín et al. [166]	Recreational training	BR	Soybean	Target: supplementation of soy proteins by vegan (V) vs. supplementation of whey proteins by omnivore (O)	Soybean protein consumption: ↑ leg lean mass, rectus femoris andvastus lateralis cross-sectional area, vastus lateralis muscle fibre No differences among groups V and O
Banaszek et al. [167]	High intensity functional training	US	Pea	Target: pea protein vs. whey protein	Pea protein consumption: ↑ muscle strength
Nieman et al. [168]	Recreational training	US	Pea	Target: pea protein isolate (PPI) vs. whey protein isolate (WPI) Control: water	Pea protein consumption: ↑ serum creatine kinase and myoglobin, AST, ALT Ø were recorded among groups PPI and WPI
Teixeira et al. [169]	Futsal	PT	Pea	Target: supplement containing pea protein (PP) vs. supplement containing whey protein (WP)	Plant based protein consumption: Ø muscle thickness of the rectus femoris; total body water; blood glucose, haematocrit, C-reactive protein, aspartate aminotransferase, alanine aminotransferase, creatine kinase, creatinine, muscle power and countermovement jump; VO2max and maximal aerobic speed Ø among groups PP and WP
Loureiro et al. [170]	Soccer	BR	Pea	Target: pea protein vs. whey protein	Pea protein consumption: ↑ ALT ↓ serum creatine kinase, AST, ALT, urea, uric acid, pain Ø level of arginine and taurine
Moon et al. [171]	Recreational training	US	Rice	Target: rice protein (RP) vs. whey protein (WP)	Rice consumption: ↑ body mass, total body water, lean mass, fat-free mass, bench press 1RM, and leg press 1RM Ø among groups RP and WP
Saracino et al. [172]	Recreational training	US	Rice Pea	Target: pea and rice protein (RP) vs. whey isolate (WI) vs. whey hydrolysate (WH) Placebo: lack of proteins	Pea and rice protein consumption: ↑ muscle soreness, creatine kinase and interleukins in blood Ø among groups RP, WI and WH.
Pinckaers et al. [173]	Recreational training	NL	Potato	Target: potato protein vs. milk protein	Potato protein consumption: ↑ mixed muscle protein synthesis
Ahmad et al. [174]	Crossfit “CINDY” exercise	MY	Maize	Target: maize beverage (MB) vs. carbohydrate-electrolyte drink (CE)	Maize consumption: ↑ total number of total number of complete set of “CINDY” exercise No significant differences among groups MB and CE in heart rate, blood lactate and rate of perceive exhaustion
Espino-González et al. [175]	Cycling	MX	Amaranth	Target: amaranth-based beverage (CHO-P; 10% and 1.5% concentrations) vs. commercial sports beverage (CHO; 6%)	Amaranth protein consumption: ↑ performance Ø among treatments

↑ increase, ↓ decrease, Ø no changes/no differences.

**Table 4:** A review of original articles devoted to the share of athletes eating products containing plant-based protein. Abbreviations: Gender: F—female, M—male, Country: BR—Brazil, CA—Canada, IE—Ireland, JP—Japan, NL—The Netherlands, PL—Poland, SI—Slovenia, US—the United States. The dot means lack of data.

References	Physical Activity	Age (Years)	Gender	Country	Results
Shaw et al. [176]	Recreational running	32.7 ± 13.5	F	CA	Considerable acceptability of supplements containing high protein concentrate from peas with low phytic acid content and high iron bioavailability
Śliż et al. [177]	Endurance athletics	39.9 ± 7	M,F	PL	Replacing meat with protein-rich plant products such as beans, chickpeas, soy, lentils, fava beans, peas at least in the majority of days a week was declared by more than 30% of participants of the investigations
Jakše, Jakše [178]	Artistic gymnastics	.	M	SI	The change in dietary habits including, among others, the consumption of plant-based proteins was confirmed by an elite female artistic gymnast
Yamada, Takeda [179]	Athletic sports, combat sports	18–23	F	JP	45.9% of athletes without PMS-related performance impairment and 39.3% of athletes with PMS-related performance impairment declared plant protein intake
Barron et al. [180]	Endurance athletics	14–23	F	US	Greater consumption of vegetable proteins such as legume and whole grain products, was declared by oligo/amenorrhoeic athletes compared to eumenorrheic athletesand non-athletes
Gillen et al. [181]	Strength, endurance and team-sport athletes	12–62	M,F	NL	Animal- and plant-based sources of protein intake were 57% and 43%, respectively
McDaid et al. [182]	Gaelic football, hurling, camogie, Athletics and triathlon, cycling, rowing	18–24	M,F	IE	The intake of plant proteins is declared only by amateur athletes
Franca et al. [183]	Judo, swimming, water polo, and artistic swimming	11–19	M,F	BR	Consumption of animal-based protein was almost four times higher than that of plant-based proteins such as legumes

**Table 5:** A review of original articles devoted to the acceptance of particular products dedicated to athletes and containing plant-based protein. Abbreviations: Gender: F—female, M—male, Country: IN—India, MY—Malaysia, US—United States. The dots mean lack of data.

References	Physical Activity	Number of Participants	Age (Years)	Gender	Country	Results
Cordelia et al. [184]	Sprint	25	17–19	M	IN	The satisfactory rating of a “Soy Protein Isolate-enriched Pregame Chocolate Bar” by runners
Sobana [185]	.	10	.	.	IN	The high acceptation for a millet-based composite sports bar
Jusoh et al. [186]	Recreational training	41	.	M,F	MY	The high rating of a recovery beverage containing proteins and carbohydrates from maize
Sommer, Vodovotz [187]	Rugby	31	.	M	US	The high rating of soybean enhanced soft pretzels designed for exercise recovery

## Data Availability

Data are contained within the article.

## Appendix A

**Table A1:** A review of patents and inventions of food products suitable for sportspeople containing amino acids, peptides and proteins derived from species representing the family *Amaranthaceae*. The invention described in the article is bolded. Country: China (CN), Colombia (CO), and Spain (ES).

Reference	First Author Affiliation	Year	Patent, Article Title	Plant Name	Component	Activity	Consistency	Form
Gazmira, Toledo [47]	ES	2016	Composition based on quinoa and gofio, as well as other components such as cocoa and rice flour	Amaranth	Protein	Nutrition	Bulk solid, solid	Powder, bar
Caceres et al. [49]	CL	2014	Method for the formulation of a gel-format foodstuff for use as a nutritional foodstuff enriched with peptides and maltodextrins obtained from quinoa flour	Quinoa	Peptides	Nutrition	Semi-solid	Gel
Yang et al. [51]	CN	2014	Chenopodium quinoa wild multi-vitamin nutrition powder processing method	Quinoa	Protein	Nutrition, physical health improvement	Bulk solid	Powder
Tao, Ting [52]	CN	2016	Quinoa nutritive meal replacement powder	Quinoa	Protein	Nutrition	Bulk solid	Powder
Paz et al. [57]	CO	2022	Protein isolate extraction from quinoa (Chenopodium Quinoa: Blanca Junin Variety) for use in high protein supplements	Quinoa	Protein isolate	Nutrition	Bulk solid	Powder

**Table A2:** A review of patents and inventions of food products suitable for sportspeople containing amino acids, peptides and proteins derived from species representing the family *Fabaceae*. The inventions described in the articles are bolded. Country: Brazil (BR), China (CN), France (FR), Germany (DE), Israel (IL), Japan (JP), Kazakhstan (KZ), The Netherlands (NL), Serbia (RS), Taiwan (TW), and The United States (US).

Reference	First Author Affiliation	Year	Patent, Article Title	Plant Name	Component	Activity	Consistency	Form
Guo et al. [53]	CN	2018	A kind of zanba, roasted qingke barley flour energy stick and preparation method thereof	Soybean	Protein	Nutrition	Solid	Stick
Hua [77]	CN	2016	Nutritious beverage containing peanut short-chain polypeptides	Peanut	Oligopeptides	Nutrition	Liquid	Beverage
Zhang et al. [80]	US	2019	Methods for the preparation of a plant protein composition	Soybean, mung bean, black bean, red bean, pea, cowpea, sword bean, hyacinth bean, kidney bean, chickpea, pigeon pea and broad bean	Protein	Nutrition	Liquid, solid	Beverage, smoothie, snack food, bakery product
Lei et al. [81]	CN	2020	Instant cereal product and preparation method thereof	Soybean, mung bean, black bean, red bean, pea, cowpea, sword bean, hyacinth bean, kidney bean, chickpea, pigeon pea and broad bean	Protein	Nutrition	Bulk solid	Instant powder
Shmulewitz, de Picciotto [82]	IL	2017	Chickpea protein products and methods of making thereof	Chickpea	Protein	Nutrition	Liquid	Emulsion
van Dijk-Ottens et al. [84]	NL	2022	Liquid nutritional composition suitable for muscle function	Soybean Pea	Protein, Protein isolate Protein	Nutrition, muscle mass increase	Liquid	Beverage
Berry et al. [85]	US	2014	Charged nutritive protein and methods	Soybean	Protein	Nutrition, muscle mass and strength increase	Liquid, semi-liquid, semi-solid, solid	Beverage, emulsion, slurry cream, paste, snack, bar
Ka et al. [88]	CN	2014	Anti-fatigue energy composition and application thereof	Soybean	Protein isolate	Nutrition, fatigue relieving	Bulk solid, solid	Granule, tablet, capsule, pills, stick
Zhanga et al. [90]	CN	2015	A kind of sport type beverage that energy is provided	Soybean	Peptides	Muscle strength increase, fatigue relieving, endurance increase	Liquid	Beverage
Liu, Wang [92]	CN	2015	Health-caring food being helpful to body strength recovery after sport	Soybean	Oligopeptide	Nutrition, muscle mass increase	Solid	Tablets, capsules, pills
Zheng et al. [93]	CN	2015	A kind of sports type nutrition bar	Soybean	Protein isolate	Nutrition	Solid	Bar
Song [95]	CN	2016	A kind of nourishing tonic for sport and containing its sport nutrition	Soybean	Protein isolate	Nutrition, muscle mass increase	Liquid	Tonic
Liang, Li [97]	US	2016	Nutritional composition and process of preparation thereof	Soybean	Protein isolate	Nutrition	Semi-liquid, bulk solid	Slurry, powder
Liu et al. [98]	CN	2016	Taurine sports drink containing soybean peptide	Soybean	Peptide	Physical health improvement, muscle mass increase	Liquid	Drink
Sun et al. [99]	CN	2016	Nutritious energy bar and preparation method thereof before one kind is run	Soybean	Protein	Nutrition	Solid	Bar
Li, Guo [100]	CN	2016	A kind of compositions for physical strength reinforcing and preparation method thereof	Soybean	Peptides, amino acids	Muscle strength increase	Liquid, bulk solid, solid	Beverage, granules, tablets, capsules
Qiu [102]	CN	2017	A kind of soya-bean polypeptides sports drink and preparation method thereof	Soybean	Peptides	Nutrition, physical health improvement	Liquid	Drink
Yuan, Qingshan [104]	CN	2017	Sweet sour bean drink and production method thereof	Kidney bean, soybean	Protein	Nutrition	Liquid	Beverage
Zhao et al. [106]	CN	2017	A kind of energy glue with anti-oxidation function and preparation method thereof	Soybean	Protein isolate	Nutrition, physical health improvement	Semi-solid	Glue
Zhu et al. [107]	CN	2018	Drinks after a kind of movement	Soybean	Protein isolate	Nutrition	Liquid	Beverage
He [109]	CN	2018	A kind of sport nutrition chewable tablets and preparation method thereof	Soybean	Protein	Nutrition	Solid	Chewable tablets
Zhou et al. [111]	CN	2018	A kind of double protein sports tonic and preparation method thereof	Soybean	Protein	Nutrition, fatigue relieving, body weight control	Liquid	Tonic
Azuma, Sumi [112]	JP	2019	Compositions for suppressing exercise-induced muscle damage	Soybean	Free amino acids, dipeptides	Muscle mass increase	Liquid, bulk solid, semi solid, solid	Beverage, suspension, emulsion, powder, granules, tablets, yogurt, gel, bar, cake
Han et al. [114]	CN	2019	Sports nutritional protein composition and preparation method thereof	Soybean	Protein isolate	Nutrition, muscle mass increase	Bulk solid	Powder
Huang et al. [115]	TW	2019	Use of green tea combined with isolated soy protein for preparing composition for improving body composition, enhancing body function or reducing fatigue after exercise capable of being used in combination with exercise training	Soybean	Protein isolate	Muscle mass and strength increase, performance increase, physical health improvement, fatigue relieving	Liquid, semi-solid, solid	Beverage, jelly, yogurt, candies, ice cream
Veloso Gonçalves Godinho [117]	BR	2019	Protein compound	Soybean	Protein isolate	Nutrition	Solid	Powder
Li et al. [120]	CN	2020	Brewing type sports nutritional meal replacement powder and preparation method thereof	Soybean	Protein isolate	Nutrition	Bulk solid	Powder
Sugita, Nashimura [122]	JP	2020	New blend sport beverage, and granular agent for preparing the same	Soybean	Protein	Fatigue relieving	Liquid, semi-liquid, semi solid, bulk solid, solid	Beverage, mouse, paste, gel, yogurt, granules, powder, tablets, capsules, cake
Xu et al. [123]	CN	2020	Double-protein sports milk and preparation method thereof	Soybean	Protein isolate	Nutrition, performance increase, fatigue relieving	Liquid	Beverage
Jovanov et al. [124]	RS	2021	High-Protein Bar as a Meal Replacement in Elite Sports Nutrition: A Pilot Study	Soybean	Protein isolate	Nutrition	Solid	Bar
Yi et al. [125]	CN	2021	Food-derived peptide and carbohydrate compositions for reducing acute exercise stress	Soybean	Peptide	Nutrition, physical health improvement	Liquid, bulk solid, solid	Beverage, powders, tablets, bars
Yamadera, Sugano [127]	JP	2021	Gelato-like food product containing protein, and method for producing the same	Soybean	Protein	Nutrition	Semi-solid	Ice dessert
Ying et al. [129]	CN	2021	Protein peptide solid beverage and preparation method and application thereof	Soybean	Peptide	Performance, endurance increase, fatigue relieving	Bulk solid	Powder
Sarsembayev et al. [131]	KZ	2022	Fermented dairy product for sports nutrition	Soybean	Protein isolate	Nutrition	Liquid	Beverage
Li et al. [133]	CN	2023	Sport nutrition protein stick and preparation method thereof	Soybean	Protein isolate	Nutrition	Solid	Bar
Wan et al. [135]	CN	2023	Liquid nutrition emulsion stabilised by alcohol washing soybean concentrated protein and preparation method thereof	Soybean	Protein concentrate	Nutrition	Liquid	Emulsion
Boursier et al. [136]	FR	2015	Assembly of at least one vegetable protein and at least one dairy protein	Pea, bean, and faba bean	Protein isolate, protein concentrate	Nutrition	Liquid, semi-solid, bulk solid	Beverage, solution, suspension, cream, powder
Budemann, Veen [138]	DE	2016	Food composition containing amino acids and cocoa	Pea, Lentil, Bean	Amino acids and/or peptides	Nutrition	Liquid, solid, bulk solid	Beverage, bar, pralines, powder
Barata et al. [139]	FR	2017	Nutritional formulations comprising a pea protein isolate	Pea	Protein isolate	Nutrition, body weight control	Liquid, semi-solid, solid	Beverage, cream, bar, cake, snacks
Hossen et al. [140]	US	2018	Processed leguminous materials	Pea	Protein concentrate, protein isolate	Nutrition	Semi-liquid	Slurry
Zhang et al. [141]	US	2018	Systems and methods using physical energy technology to produce non-dairy protein base and value-added utilisation of the co-product	Pea	Protein	Nutrition	Semi-liquid	Slurry
Liang et al. [143]	CN	2021	Black beanwhey double protein sports nutrition powder useful for regulating digestion and absorption of nutrients, comprises black bean protein powder, and whey protein powder	Black bean	Protein	Nutrition, physical health improvement, body weight control	Bulk solid	Powder
Abdel-Salam F.F. et al. [144]	EG	2022	Formulation and Evaluation of High Energy-protein Bars as a Nutritional Supplement for Sports Athletics	Kidney bean	Protein	Nutrition	Solid	Bar

**Table A3:** A review of patents and inventions of food products suitable for sportspeople containing amino acids, peptides and proteins derived from species representing the family *Poaceae*. Country: Belgium (BE), China (CN), Finland (FI), The Russian Federation (RU), and The United States (US).

Reference	First Author Affiliation	Year	Patent, Article Title	Plant Name	Component	Activity	Consistency	Form
Awadh et al. [146]	FI	2022	Instant colloidal whole oat flour applications	Oat	Protein	Nutrition	Bulk solid	Powder
Janow [147]	US	2014	Rice protein supplement and methods of use thereof	Rice	Protein isolate	Nutrition	Bulk solid	Powder bar
Kovalchuk [148]	RU	2019	Whole-grain protein chips and their production method	Maize Rice	Protein	Nutrition	Solid	Chips
Xiaomeng [149]	CN	2015	Exercise-fatigue-resistant nutrition protein bar and method for preparing same	Common wheat	Protein	Nutrition, fatigue relieving	Solid	Bar
Hongjun [150]	CN	2016	Drink for enhancing immunity and improving athletic ability	Common wheat	Oligopeptides	Physical health improvement, fatigue relieving, performance increase	Liquid	Beverage
Marichez [151]	BE	2016	Nutritional composition rich in wheat protein	Common wheat	Protein hydrolysate	Nutrition	Liquid, semi-solid, bulk solid, solid	Beverage, gel, powder, cream, biscuit
Liu, Ke [152]	CN	2019	Protein composition for rapid energy replenishment	Common wheat	Oligopeptide, amino acids	Nutrition	Solid	Rod
Zhang et al. [153]	CN	2014	Corn germ-containing sports chocolates and preparation method thereof	Maize	Peptides	Nutrition	Solid	Chocolates
Anonymous [154]	CN	2017	A kind of teenager’s corn peptide sports beverage and preparation method thereof	Maize	Peptides	Nutrition, mental and physical health improvement, fatigue relieving	Liquid	Beverage
Binga et al. [155]	CN	2018	A kind of sports type nutritional supplement and preparation method thereof	Maize	Peptides	Nutrition, muscle mass increase, control of body weight	Bulk solid	Powder

**Table A4:** A review of patents and inventions of food products suitable for sportspeople containing amino acids, peptides and proteins derived from species representing the families *Cannabaceae*, *Polygonaceae* and *Solanaceae*. Country: China (CN), and The United States (US).

Family	Reference	First Author Affiliation	Year	Patent, Article Title	Plant Name	Component	Activity	Consistency	Form
*Cannabaceae*	Cao et al. [72]	CN	2019	Spasm-resisting sports beverage and preparation method thereof	Hemp	Protein	Physical health improvement	Liquid	Beverage
*Polygonaceae*	Zuo et al. [156]	CN	2019	Composite black Jerusalem artichoke sports tea beverage and preparation method thereof	Tartary buckwheat	Protein	Nutrition, fatigue relieving	Liquid	Beverage
*Solanaceae*	Bolscheid et al. [157]	US	2018	Potato protein powders	Potato	Protein, amino acids	Nutrition	Bulk solid	Powder

**Table A5:** A review of patents and inventions of food products suitable for sportspeople containing amino acids, peptides and proteins derived from species representing different botanical families. The inventions described in the articles are bolded. Country: Australia (AU), Brazil (BR), Canada (CA), China (CN), Denmark (DK), India (IN), Italy (IT), Japan (JP), The Netherlands (NL), Norway (NO), Poland (PL), Slovenia (SI), Sweden (SE), Switzerland (CH), and The United States (US).

Reference	First Author Affiliation	Year	Patent, Article Title	Plant Name	Component	Activity	Consistency	Form
Methner et al. [48]	DE	2019	Sports drinks and procedures for their production	Soybean, common wheat, rye, oats, beans, peas, quinoa, amaranth	Protein	Nutrition	Liquid	Beverage
Cain, Milazzo [50]	US	2014	Nutraceutical formulation	Hemp, quinoa, soybean, sweet potato	Protein	Muscle mass increase	Bulk solid	Powder
Zwijsen et al. [54]	NL	2018	Nutritional compositions for musculoskeletal support for athletes	Soybean, canola, rapeseed, common wheat, rice, quinoa, pea, maize	Protein	Nutrition, muscle strength	Liquid, solid	Beverage, shake, powder
Rischbieter, Biondo [55]	BR	2019	High-energy food supplement based on inverted sugars and ergogenic products for use in physical activities and their production process	Soybean, pea, lentil, chickpea, quinoa, rice	Protein	Nutrition	Liquid, semi-solid	Syrup, gel, paste
Bertocco et al. [56]	US	2020	Nutritional plant-based protein compositions with high digestibility	pearice, flaxseed, quinoa	Protein, protein isolate, protein concentrateprotein	Physical health improvement	Liquid, solid	Beverage, powder, granules, tablets, pellets
Filip, Vidrih [58]	SI	2015	Amino Acid Composition of Protein-Enriched Dried Pasta: Is It Suitable for a Low-Carbohydrate Diet?	Pea, Spinach	Protein isolate Protein	Nutrition	Solid	Pasta
Li [59]	CN	2017	Spinach sports beverage and preparation method thereof	Spinach Pea, peanut	Protein isolate Protein	Fatigue relieving	Liquid	Beverage
Vadakkemuri et al. [60]	IN	2015	Optimised nutrient food	Soybean, goa beans, hemp, water melon, pumpkin, sesame, pistachios, gingili, poppy, flax, ground nut, water melon	Protein	Nutrition, physical health improvement, performance increase	Bulk solid	Powder
Braun et al. [61]	CH	2016	Heat sterilised high protein compositions with hydrolysed protein from a continuous process with at least one endopeptidase	Soybean pea, sunflower, potato	Protein and protein isolate Protein	Nutrition	Liquid, bulk solid	Beverage, powder
Mathisen, Mathisen [62]	NO	2020	Omega-3 beverage	Sunflower, chia, lupin and coconut	Protein Peptides	Physical health improvement	Liquid	Beverage
Kvistgaard, Søndergaard [63]	DK	2021	Food comprising isosteviol and uses thereof	Alfalfa, cotton, oil palm, coconut, sesame, crambe, clover, bean, pea, chickpea, lentil, lupin, mesquite, carob, peanuts, tamarind, maize, sunflower, hemp, soybean	Protein	Muscle mass increase, endurance increase,	Liquid, semi-liquid, semi-solid, solid	Beverage, slurry, gel, grains, capsule, powder
Zhang et al. [64]	US	2021	Powdered composition	Pea, lentil, rice, potato, chickpea, fava bean, mung bean, sunflower, pumpkin, flax, chia, canola, lupine, alfalfa, Meringa	Protein	Nutrition	Liquid, semi-solid, solid	Beverage, ice creams, sorbets, yogurts bar, cake, powder
Breuille et al. [65]	CH	2014	Methods for enhancement of muscle protein synthesis	Soybean, Pea, wheat, rice, canola, oat, rice, peanut	Protein protein, protein isolate protein	Nutrition, muscle mass increase	Liquid, semi-solid, bulk solid, solid	Emulsion, gel, yogurt, powder, capsules, tablets
Jenkins [66]	US	2014	Protein beverage and method of making same	Soybean, rice, pea, canola, wheat, hemp, flax	Protein	Muscle mass increase	Liquid	Beverage
Phillips et al. [67]	CA	2018	Multi-nutrient composition	Soybean, pea, bean, maize, rice, canola, peanut, lentil	Protein	Nutrition, muscle strength, mental and physical health improvement	Liquid, semi-liquid semi-solid, solid	Beverage, emulsion, suspension, gel, bar, powder, pill, tablet, capsule
Franse et al. [68]	NL	2022	Protein bar	Pea Rapeseed	Protein isolate	Nutrition	Solid	Bar
Voudouris et al. [69]	NL	2022	Food formulation with high protein content	Chickpea, canola, soybean	Protein isolate	Nutrition, post-exercise muscle recovery	Liquid, semi-liquid	Beverage, slurry
Bonetto [70]	IT	2020	Food supplement for sports use	Moringa, goji, pea, rice	Protein	Endurance	Solid	Powder
Davie [72]	AU	2017	A nutritional supplement	Soybean, pea, rice, hemp	Protein concentrates and isolate	Nutrition, muscle mass increase	Liquid, bulk solid	Beverage, powder
Komorowski [73]	AU	2023	Chromium containing compositions for improving health and fitness	Soybean, pea, hemp, rice	Protein	Muscle mass and strength improvement, endurance increase	Liquid, solid	Concentrate, powder
Yago et al. [74]	JP	2020	Composition for sympathetic nerve activation	Soybean, wheat, barley, pea, maize, potato, sweet potato	Peptides	Physical health improvement	Liquid, solid	Beverage, syrup, emulsion, powders, granules, tablets and capsules
Li [75]	CN	2017	A kind of peptide meal powder and preparation method thereof	Kidney bean, maize, rice, Chinese yam, matrimony vine, walnut	Peptides	Nutrition	Bulk solid	Powder
Beliciu et al. [76]	US	2018	Ready-to-drink plant protein beverage product and methods for making same	Chickpea, lentil, lupin, pea, sacha inchirice, wheat	Protein, protein isolate	Nutrition	Liquid	Beverage
Liu et al. [78]	CN	2016	Clean preparation method for plant polypeptide, protein	Soybean, peanut, maize, common wheat	Peptides	Nutrition, muscle recovery	Liquid	Beverage
Sheng et al. [79]	CN	2021	Preparation method of moringa oleifera and walnut protein energy bar	Soybean, walnut, peanut	Protein	Nutrition	Solid	Bar
Oliver et al. [83]	CA	2019	Plant-based whey protein powder replacement	Fava bean chickpea	Protein concentrates and isolate, protein concentrate	Nutrition	Solid	Powder
Burke et al. [86]	AU	2014	Method of enhancing muscle protein synthesis	Soybean, pea, common wheat, rice	Protein, protein isolate, protein hydrolysate, amino acids Protein	Muscle mass increase	Semi-solid bulk solid	Gel, powder
Jeppensen Bendix, Lavrsen [87]	DK	2014	Compositions for use in restoring muscle glycogen and, or muscle mass	Soybean, pea, rice, maize, common wheat	Protein and protein isolate and concentrate protein	Muscle mass increase	Liquid, semi-solid	Juice, nectar, drink cream, jam, gels, yoghurt, pudding, jelly
Roberts et al. [89]	US	2014	Physical optimisation beverage	Soybean, common wheat, rice, maize	Protein	Muscle mass increase, fatigue relieving, performance increase, physical health improvement	Liquid	Beverage
Fu, Xia [91]	CN	2015	Microcapsule powder for supplementing omega-3 fatty acid and oligopeptide and preparation method	Soybean, maize	Protein and oligopeptide concentrate	Nutrition	Bulk solid	Powder
Shi et al. [94]	CN	2015	Natural functional beverage composition capable of improving efficiency during movement training and application of natural functional beverage composition	Soybean, potato	Protein isolate	Performance increase, physical health improvement	Liquid	Beverage
Honda et al. [96]	JP	2016	Muscle enhancing composition	Soybean, common wheat	Protein, peptides	Muscle mass and strength increase	Liquid, semi-solid, bulk solid, solid	Drinks, jellies granules, tablets, capsules, pills
Li et al. [101]	CN	2017	A kind of composition for strengthening body movement ability and alleviating sports fatigue	Soybean, maize, common wheat	Peptides	Nutrition, fatigue relieving	Liquid, bulk solid	Beverage, powder
He et al. [103]	CN	2017	A kind of plant energy rod suitable for sport people and preparation method thereof	Soybean quinoa	Protein, peptides peptides	Nutrition	Solid	Stick
Yao et al. [105]	CN	2017	One kind is refreshed oneself anti-fatigue solid beverage	Soybean, maize	Peptides	Fatigue relieving, physical health improvement	Liquid, bulksolid	Beverage, powder
Guo et al. [108]	CN	2018	Full quinoa activity peptide nutrient food and preparation method thereof	QuinoaSoybean, fava bean, pea, walnut	Peptide, protein isolate	Nutrition	Liquid, bulk solid, solid	Beverage, Powder, Capsule, Tablet
Öste et al. [110]	SE	2018	Food supplement and composition for treating the metabolic syndrome	Soybean, pea, oat, barley	Peptides and amino acids	Nutrition, physical health improvement, body weight control	Liquid, semi-liquid semi-solid, solid	Beverage, emulsion, porridge, cream, gel, paste, ice-cream, powder
Amyx et al. [113]	US	2019	Sports and nutritional supplement formulations	Soybean, common wheat, rice, maize	Protein and protein concentrate Protein	Nutrition, muscle strength, endurance increase	Liquid, semi-solid, solid	Beverage, syrup, gel, capsule, tablet, bar, powder
Toyooka et al. [116]	JP	2019	Gel-like food composition	Soybean, maize, common wheat	Protein and Peptides	Nutrition, fatigue relieving	Semi-solid	Gel
Wu, Zhang [118]	CN	2019	A kind of oral polypeptide powder and its preparation method and application	Soybean, rice, common wheat	Peptide	Nutrition	Solid	Powder
Elonis et al. [119]	US	2020	Meal in a pill and methods for the same	Soybean, pea Rice, common wheat	Protein isolate Peptides and amino acids	Nutrition	Solid	Pill
Luo et al. [121]	CN	2020	Sports nutritional composition and application thereof	Soybean, maize	Peptides Oligopeptides	Nutrition	Liquid, solid	Beverage, powder
Tan [126]	JP	2021	Beverage	Soybean, maize	Protein	Physical health improvement	Liquid	Beverage
Yao et al. [128]	CN	2021	Bovine bone peptide and salmon collagen composite repair protein stick and preparation method thereof	Soybean, maize	Protein	Muscle mass increase, relieving fatigue	Solid	Stick
Małecki et al. [130]	PL	2022	Physicochemical, nutritional, microstructural, surface and sensory properties of a model high-protein bars intended for athletes depending on the type of protein and syrup used	Rice Pea, soybean	Protein concentrate Protein isolate	Nutrition	Solid	Bar
Liu et al. [132]	CN	2023	Protein stick for supplementing meal replacement and fat reduction functions of women in exercise and preparation method thereof	Soybean Rice, pea Maize	Protein, peptides Protein Peptides	Nutrition, physical health improvement	Solid	Stick
Song et al. [134]	CN	2023	Water-soluble dietary fibre-plant peptide anti-fatigue solid beverage and preparation method thereof	Soybean, walnut, red date	Peptides	Nutrition, fatigue relieving	Solid	Powder
Lis et al. [137]	FR	2015	Novel non-allergenic snacks containing vegetable protein	Alfalfa, clover, lupine, pea, beans, beans, faba beans, lens, wheat, oats, rye, barley, maize, sorghum, rice	Protein	Nutrition	Solid	Snack
De Vel et al. [142]	BE	2021	Plant-based protein powders for beverages	Pea, rice	Protein hydrolysate	Muscle mass increase	Solid	Powder
Kosovan et al. [145]	RU	2014	Bakery products preparation method	Flax, sesame	Protein	Nutrition	Solid	Bread
